# Association between metabolic body composition status and vitamin D deficiency: A cross-sectional study

**DOI:** 10.3389/fnut.2022.940183

**Published:** 2022-07-28

**Authors:** Yi-Chuan Chen, Wen-Cheng Li, Pin-Hsuan Ke, I-Chun Chen, Wei Yu, Hsiung-Ying Huang, Xue-Jie Xiong, Jau-Yuan Chen

**Affiliations:** ^1^Department of Family Medicine, Chang Gung Memorial Hospital, Linkou Branch, Taoyuan, Taiwan; ^2^College of Medicine, Chang-Gung University, Taoyuan, Taiwan; ^3^Department of Health Management, Xiamen Chang Gung Hospital Hua Qiao University, Xiamen, China; ^4^Department of Pulmonary and Critical Care Medicine, Xiamen Chang Gung Hospital Hua Qiao University, Xiamen, China; ^5^Department of Oncology, Xiamen Chang Gung Hospital Hua Qiao University, Xiamen, China

**Keywords:** metabolic body composition, obesity, vitamin D deficiency, inflammatory marker, cardiometabolic marker

## Abstract

This study aimed to investigate the risk of vitamin D deficiency in a relatively healthy Asian population, with (i) metabolically healthy normal weight (MHNW) (homeostasis model assessment-insulin resistance [HOMA-IR] < 2. 5 without metabolic syndrome [MS], body mass index [BMI] < 25), (ii) metabolically healthy obesity (MHO) (HOMA-IR < 2.5, without MS, BMI ≥ 25), (iii) metabolically unhealthy normal weight (MUNW) (HOMA-IR ≥ 2.5, or with MS, BMI < 25), and (iv) metabolically unhealthy obesity (MUO) (HOMA-IR ≥ 2.5, or with MS, BMI ≥ 25) stratified by age and sex. This cross-sectional study involved 6,655 participants aged ≥ 18 years who underwent health checkups between 2013 and 2016 at the Chang Gung Memorial Hospital. Cardiometabolic and inflammatory markers including anthropometric variables, glycemic indices, lipid profiles, high-sensitivity C-reactive protein (hs-CRP), and serum 25-hydroxy vitamin D levels, were retrospectively investigated. Compared to the MHNW group, the MHO group showed a higher odds ratio (OR) [1.35, 95% confidence interval (CI) 1.05–1.73] for vitamin D deficiency in men aged < 50 years. By contrast, in men aged > 50 years, the risk of vitamin D deficiency was higher in the MUO group (OR 1.44, 95% CI 1.05–1.97). Among women aged < and ≥ 50 years, the MUO group demonstrated the highest risk for vitamin D deficiency, OR 2.33 vs. 1.54, respectively. Our study revealed that in women of all ages and men aged > 50 years, MUO is associated with vitamin D deficiency and elevated levels of metabolic biomarkers. Among men aged < 50 years, MHO had the highest OR for vitamin D deficiency.

## Introduction

The prevalence of obesity has been increasing worldwide. Obesity-related disorders have been widely studied. Research has focused on visceral fat accumulation, which is recognized as a cardiometabolic risk factor (1). Adipocyte hypertrophy results in unbalanced blood flow, local hypoxia, inflammatory macrophage infiltration, increased synthesis, and release of pro-inflammatory mediators [such as tumor necrosis factor-alpha [TNF-α], interleukin [IL]-6, and IL-8], and tissue inflammation (2, 3). Adipokines such as TNF and IL-6, which are secreted from visceral fat, may contribute to the development of atherosclerosis (4, 5). Body mass index (BMI) is a convenient tool for assessing the extent of overweight and obesity. However, BMI has limitations in the evaluation of body composition and metabolic status (6–8).

Some obese individuals appear to be protected from the development of metabolic disturbances or complications; thus, they are referred to as metabolically healthy obesity (MHO) (9). Individuals with MHO have lower visceral fat values than individuals with a similar body fat percentage (10). In addition, despite having excessive body weight, individuals with MHO demonstrate normal blood pressure, lipid profile, insulin sensitivity, inflammatory markers such as C-reactive protein (CRP) (11–13), and favorable levels of liver enzymes, which may reflect lower liver fat content (14) without significantly increased risks of diabetes and cardiovascular diseases (15, 16). Several mechanisms have been hypothesized to explain MHO. For example, high mitochondrial transcription and low inflammation levels in subcutaneous adipose tissue are associated with lower liver fat and MHO levels (17). In addition, differences in visceral fat accumulation, birth weight, adipose cell size, gene expression encoding markers of adipose cell differentiation (11) and lipolysis (18) were suggestive of MHO phenotype development.

By contrast, some individuals with normal weight but metabolic disturbances or complications were defined as metabolically unhealthy normal weight (MUNW) groups. Such individuals might be characterized by higher body fat percentage, visceral fat and insulin levels, increased adipocyte size, and predisposition to type 2 diabetes mellitus (T2DM), hyperlipidemia, and cardiovascular diseases compared with patients with a similar BMI (19–24). Central fat distribution, lower physical activity energy expenditure, and lower peak oxygen uptake appeared to be predisposing factors for MUNW. In addition, a cognitive attitude toward food and lifestyle plays a role in insulin sensitivity in MUNW (25).

Vitamin D involves in a variety of processes in human body. In addition to calcium (Ca) homeostasis and bone metabolism, vitamin D plays an important role in multiple organs and has many physiological functions (26). Recent studies suggest that low vitamin D levels are a risk factor not only for osteoporosis, sarcopenia, and frailty, but also for infection, autoimmune diseases, and other cardiometabolic diseases, such as hypertension (27, 28), diabetes (29) and metabolic syndrome (30–37). Vitamin D is mediated by the vitamin D receptor (VDR), which regulates the transcription of several target genes (38). VDR has been identified in a large variety of cell types, including monocytes, cardiomyocytes, pancreatic beta cells, vascular endothelial cells, neurons, immune cells, and osteoblasts (39). Recent studies have demonstrated that VDR and vitamin D-metabolizing enzymes are expressed in adipocytes (40).

Evidence has shown that vitamin D inhibits the expression of adipogenic transcription factor genes, leading to a significant reduction in lipid accumulation and adipocyte apoptosis (2, 41). Obese individuals tend to have lower vitamin D levels (42–46), predisposing them to the development of comorbidities. Increased sequestration by the white adipose tissue reduces vitamin D bioavailability (47, 48). Vitamin D deficiency is also associated with the dysregulation of white adipose tissue and blood levels of inflammatory factors, including CRP and IL-10 (2). Several cross-sectional and cohort studies have found a positive correlation between vitamin D levels, beta cell function (49), and insulin sensitivity. Patients with low vitamin D levels appear to have a high risk of insulin resistance. The potential role of vitamin D deficiency in insulin resistance has been associated with inherited gene polymorphisms, including vitamin D-binding protein, vitamin D receptor, and the vitamin D 1 alpha-hydroxylase gene (30, 50–54).

After a thorough literature search, we found that the association between vitamin D deficiency and metabolic body composition status remains controversial. Furthermore, little has been reported about cardiometabolic markers among the different metabolic phenotypes with respect to sex and age (55). Since male and female have different features of adiposity distribution, which may affect vitamin D bioavailability, coupled with changes in metabolism due to the loss of protection from hormones after menopause, we aimed to compare the impact of metabolic phenotypes on the risk of vitamin D deficiency stratified by sex and age. We hypothesized that (i) metabolically unhealthy obesity (MUO) is an independent risk factor for vitamin D deficiency; (ii) in male participants of all ages and female participants aged > 50 years, cardiometabolic markers have incremental trends among the healthy, MHO, MUNW, and MUO groups.

## Materials and methods

### Study design and participants

We retrospectively obtained data from adult participants (age ≥ 18 years) who underwent health checkups between 2013 and 2016 at Chang Gung Memorial Hospital. The exclusion criteria were as follows: (i) fasting < 12 h; (ii) pregnancy; (iii) conditions that may affect the metabolic status, such as hyperthyroidism or hypothyroidism, malignancy, chronic hepatitis, liver cirrhosis, hypothalamic disease, pituitary gland, or adrenal gland diseases; (iv) parathyroid gland disease or intake of medications that may affect vitamin D level; (v) high sensitivity (hs)-CRP > 10 mg/L, which may indicate acute infection status; and (vi) participants with incomplete data and history. In total, 6,655 participants were included in the analysis. Informed consent was not obtained because all data were accessed anonymously in the setting of retrospective records.

### Data collection

Trained nurses used a standardized questionnaire to collect information on patients' medical and personal histories. Completion of the questionnaire was followed by anthropometric measurement, including body weight (kg), height (centimeter, cm), waist circumference (cm), and blood pressure (mmHg). Body height and weight were measured using calibrated meters and scales, according to a standardized protocol. BMI was calculated as body weight divided by the square of body height (kg/m^2^). Waist circumference was measured midway between the lowest rib and iliac crest. Blood pressure was measured using an automated sphygmomanometer three times after the participants were seated for at least 15 min.

Laboratory data included total cholesterol (TC), low-density lipoprotein-cholesterol (LDL-C, mmol/L), high-density lipoprotein-cholesterol (HDL-C, mmol/L), triglyceride (TG, mmol/L), fasting blood glucose (FBG, mmol/L), hs-CRP (μg/mL), and insulin, which were determined using enzymatic, spectrophotometric, or colorimetric methods. Between 2013 and 2014, serum 25(OH)D levels were quantitatively determined using an electrochemiluminescence assay (ECLIA) performed on a Roche Cobas E601 immunoassay system (Roche Diagnostics, Mannheim, Germany); the unit of measurement was ng/mL. After 2015, it was determined using a chemiluminescent microparticle immunoassay (CMIA) on an Abbott I2000SR immunoassay system (Abbott Diagnostics, Illinois, USA), and the measurement unit was nmol/L. All the data were entered into an electronic database under strict quality control.

Participants who fulfilled at least three of the five criteria described by the Third Adult Treatment Panel (ATP III) of the National Cholesterol Education Program (NCEP) were defined as having metabolic syndromes. The five factors are high blood pressure (systolic blood pressure ≥ 130 mmHg and diastolic pressure ≥ 85 mmHg), under treatment, or already diagnosed with hypertension); high serum TG (≥ 1.7 mmol/L or under treatment); decreased HDL-C (<1.03 mmol/L for males and < 1.29 mmol/L for females or under treatment); hyperglycemia (FBG ≥ 5.6 mmol/L, under treatment, or previously diagnosed with diabetes mellitus); and abdominal obesity (waist circumference ≥ 90 cm for males and ≥ 80 cm for females).

### Definition of homeostasis model assessment-insulin resistance

HOMA-IR index was used to quantify the extent of insulin resistance. The cutoff value of HOMA-IR as an indicator of metabolic syndrome was based on two recent studies in Asian populations (26, 27). The HOMA-IR index formula was as follows:


$$\text{fasting insulin (mIU/L) }\times \frac{FBG(mmol/L\;)}{22.5}$$

### Phenotypes of metabolic body composition status

All participants were categorized into four metabolic phenotypes:

(i) metabolically healthy normal weight (MHNW) (HOMA-IR <2.5, without MS, BMI <25), (ii) MHO (HOMA-IR <2.5, without MS, BMI ≥ 25), (iii) MUNW (HOMA-IR ≥ 2.5 or with MS, BMI <25), and (iv) MUO (HOMA-IR ≥ 2.5 or with MS, BMI ≥ 25).

### Definition of vitamin D deficiency

A serum vitamin D level < 20 ng/mL is defined as vitamin D deficiency according to the Endocrine Society Clinical Practice Guidelines (56).

### Statistical analysis

The mean ± standard deviation (SD) was used for continuous variables and the number (%) was used for categorical variables. The independent *t*-test and chi-square tests were used to compare differences between sexes for continuous and categorical variables, respectively. Analysis of variance and chi-square tests were used to compare the differences among different metabolic states (MHNW, MHO, MUNW, and MUO) for continuous and categorical variables, respectively. Additionally, linear contrast in the analysis of variance and the Cochran-Armitage test were used to determine the linear trend across metabolic states for continuous and categorical variables, respectively. Bonferroni *post hoc* comparisons were performed for pairwise analyses of the study groups. Multiple logistic regression models were used to explore the relationship between the metabolic phenotypes and vitamin D deficiency. We chose the mean arterial pressure, TG/HDL-C ratio, and hs-CRP level as covariates. Sex, age, and HOMA-IR were grouped variables; thus, they were not adjusted for. Neither were FBG and insulin levels adjusted for because they were highly correlated with HOMA-IR. Metabolic body composition was a variable of interest; therefore, BMI and waist-to-height ratio were not adjusted for. The LDL-C level was not adjusted for because of its collinearity with TC. All statistical analyses were conducted using International Business Machine (IBM) Statistical Product and Service Solutions Statistics (SPSS, IBM Corp., Armonk, NY, USA). Statistical significance was set at a *P*-value < 0.05.

## Results

### Demographics of the study participants

A total of 6,655 participants were enrolled in this study. The main characteristics of the study participants, stratified by age (< 50 and ≥ 50 years), are shown in Table 1. In the < 50 years group (*n* = 2,589), the mean age and BMI of the male participants were slightly higher than that of the female participants. Mean arterial pressure (MAP), TC, TG, LDL-C, hs-CRP, insulin, and HOMA-IR were significantly higher in men than that in women (*P* < 0.001). The proportion of MHO and MUO were higher in men than that in women (30.2% vs. 13.0% and 17.3% vs. 4.0%, respectively). The prevalence of vitamin D deficiency was significantly higher in women than that in men (35.5% vs. 31.2%, *P* = 0.025; Table 1).

**Table 1:** Main characteristics of the participants by age and sex.

	<**50 years old**				≥**50 years old**			
**Characteristics**	**Total** **(*****N*** = **2,589)**	**Men** **(*****n*** = **1,629)**	**Women** **(*****n*** = **960)**	***P*** **-value**	**Total** **(*****N*** = **4,066)**	**Men** **(*****n*** = **2,210)**	**Women** **(*****n*** = **1,856)**	***P*** **-value**
Age, years	40.9 ± 6.0	41.0 ± 5.8	40.7 ± 6.2	0.232	58.4 ± 6.7	58.6 ± 6.9	58.2 ± 6.5	0.097
BMI (kg/m^2^)	23.8 ± 3.5	24.8 ± 3.4	22.2 ± 3.0	<0.001	24.2 ± 3.2	24.3 ± 3.1	24.2 ± 3.2	0.496
Waist-to-height ratio	0.50 ± 0.05	0.51 ± 0.05	0.48 ± 0.05	<0.001	0.52 ± 0.05	0.52 ± 0.05	0.53 ± 0.05	<0.001
Mean arterial pressure (mmHg)	86.1 ± 12.8	89.8 ± 12.1	79.8 ± 11.3	<0.001	91.4 ± 13.3	92.2 ± 13.0	90.4 ± 13.5	<0.001
Fasting glucose (mmol/L)	5.4 ± 1.3	5.5 ± 1.6	5.11 ± 0.67	<0.001	5.7 ± 1.6	5.8 ± 1.8	5.6 ± 1.3	<0.001
Total cholesterol (mmol/L)	5.2 ± 1.0	5.3 ± 1.0	4.9 ± 0.9	<0.001	5.4 ± 1.0	5.3 ± 1.0	5.5 ± 1.0	<0.001
TG (mmol/L)	1.5 ± 1.3	1.9 ± 1.5	0.95 ± 0.78	<0.001	1.5 ± 1.1	1.6 ± 1.3	1.35 ± 0.89	<0.001
LDL-C (mmol/L)	3.23 ± 0.89	3.40 ± 0.91	2.94 ± 0.78	<0.001	3.42 ± 0.88	3.39 ± 0.88	3.46 ± 0.87	0.008
HDL-C (mmol/L)	1.27 ± 0.31	1.17 ± 0.27	1.44 ± 0.31	<0.001	1.29 ± 0.32	1.20 ± 0.30	1.39 ± 0.31	<0.001
TG / HDL-C	1.4 ± 1.6	1.8 ± 1.8	0.74 ± 0.93	<0.001	1.3 ± 1.3	1.5 ± 1.5	1.09 ± 0.93	<0.001
hs-CRP (μg/mL)	1.7 ± 3.9	2.1 ± 4.5	1.1 ± 2.6	<0.001	2.4 ± 6.2	2.6 ± 6.8	2.1 ± 5.3	0.019
Insulin (mIU/L)	7.1 ± 4.0	7.6 ± 4.4	6.3 ± 3.0	<0.001	6.6 ± 4.2	6.4 ± 4.2	6.9 ± 4.1	<0.001
HOMA-IR	1.7 ± 1.2	1.9 ± 1.4	1.46 ± 0.79	<0.001	1.7 ± 1.4	1.7 ± 1.5	1.7 ± 1.3	0.578
Metabolic phenotypes				<0.001				0.049
MHNW, *n* (%)	1,543 (59.6)	790 (48.5)	753 (78.4)		2,255 (55.5)	1,219 (55.2)	1,036 (55.8)	
MHO, *n* (%)	617 (23.8)	492 (30.2)	125 (13.0)		1,127 (27.7)	625 (28.3)	502 (27.0)	
MUNW, *n* (%)	109 (4.2)	65 (4.0)	44 (4.6)		215 (5.3)	99 (4.5)	116 (6.3)	
MUO, *n* (%)	320 (12.4)	282 (17.3)	38 (4.0)		469 (11.5)	267 (12.1)	202 (10.9)	
25(OH)D (ng/mL)	28.8 ± 14.7	30.0 ± 15.5	26.8 ± 13.1	<0.001	33.9 ± 16.8	35.9 ± 18.3	31.5 ± 14.4	<0.001
Vitamin D deficiency, *n* (%)	850 (32.8)	509 (31.2)	341 (35.5)	0.025	955 (23.5)	501 (22.7)	454 (24.5)	0.180

BMI, body mass index; TG, Triglycerides; LDL-C, low-density lipoprotein-cholesterol; HDL-C, high-density lipoprotein-cholesterol; hs-CRP, high-sensitivity C-reactive protein; HOMA-IR, Homeostasis Model Assessment-Insulin Resistance; 25(OH)D, 25-OH-Vitamin D; MHNW, Metabolically healthy normal weight; MHO, metabolically healthy obesity; MUNW, metabolically unhealthy normal weight; MUO, metabolically unhealthy obesity.

In the ≥ 50 years group (*n* = 4,066), the mean ± SD age of the participants was 58.6 ± 6.9 for men and 58.2 ± 6.5 for women. The mean ± SD BMI was 24.3 ± 3.1 for men and 24.2 ± 3.2 for women. No significant difference was observed in the mean ± SD age or BMI between men and women (Table 1).The MAP, FBG, TG, and hs-CRP levels were significantly higher in men than that in women, while TC, LDL-C, and insulin levels were significantly higher in women. HOMA-IR levels and vitamin D deficiency were not significantly different between the sexes. The proportion of MHO and MUO were higher in male than that in female. Vitamin D deficiency was observed in 22.7% of men and 24.5% of women, without a significant difference (*P* = 0.18).

### Characteristics of men according to metabolic phenotypes

The baseline characteristics of men according to metabolic phenotypes stratified by age are presented in Table 2. The male participants were classified into four groups: MHNW, MHO, MUNW, and MUO.

**Table 2:** Baseline characteristics of men according to metabolic phenotypes stratified by age.

**Characteristics**	**MHNW**	**MHO**	**MUNW**	**MUO**	***P*** **-value**	***P*** **-trend**
Men <50 years old (*n* = 1,629)						
Number	790	492	65	282		
Age, years	40.9 ± 5.9	41.5 ± 5.6	42.3 ± 5.4	40.4 ± 6.2	0.015	0.653
BMI (kg/m^2^)	22.2 ± 2.0	27.1 ± 1.8^a^	23.3 ± 1.4^a, b^	28.4 ± 2.6^a, b, c^	<0.001	<0.001
Waist-to-height ratio	0.48 ± 0.04	0.54 ± 0.03^a^	0.51 ± 0.03^a, b^	0.56 ± 0.04^a, b, c^	<0.001	<0.001
Mean arterial pressure (mmHg)	85.8 ± 10.9	92.3 ± 11.9^a^	91.4 ± 12.1^a^	96.5 ± 11.7^a, b, c^	<0.001	<0.001
Fasting glucose (mmol/L)	5.3 ± 1.1	5.2 ± 0.53	6.9 ± 3.3^a, b^	6.5 ± 2.5^a, b^	<0.001	<0.001
Total cholesterol (mmol/L)	5.22 ± 0.95	5.31 ± 1.03	5.47 ± 1.02	5.54 ± 1.02^a, b^	<0.001	<0.001
TG (mmol/L)	1.5 ± 1.0	2.1 ± 1.7^a^	2.2 ± 1.5^a^	2.6 ± 1.7^a, b^	<0.001	<0.001
LDL-C (mmol/L)	3.33 ± 0.85	3.41 ± 0.92	3.59 ± 0.99	3.53 ± 1.02^a^	0.006	0.001
HDL-C (mmol/L)	1.25 ± 0.30	1.11 ± 0.21^a^	1.13 ± 0.31^a^	1.06 ± 0.20^a^	<0.001	<0.001
TG / HDL-C	1.3 ± 1.1	2.0 ± 2.3^a^	2.2 ± 1.8^a^	2.6 ± 1.9^a, b^	<0.001	<0.001
hs-CRP (μg/mL)	1.6 ± 3.8	2.3 ± 4.4^a^	2.2 ± 5.6	3.4 ± 5.8^a, b^	<0.001	<0.001
Insulin (mIU/L)	5.3 ± 2.03	7.1 ± 1.96^a^	12.3 ± 6.4^a, b^	13.7 ± 5.0^a, b, c^	<0.001	<0.001
HOMA-IR	1.23 ± 0.51	1.64 ± 0.47^a^	3.5 ± 2.1^a, b^	3.8 ± 1.8^a, b^	<0.001	<0.001
25(OH)D (ng/mL)	31.4 ± 16.7	28.2 ± 14.4^a^	31.5 ± 15.2	29.1 ± 13.6	0.002	0.352
Vitamin D deficiency, *n* (%)	234 (29.6)	176 (35.8)	20 (30.8)	79 (28.0)	0.071	0.782
Men ≥50 years old (*n* = 2,210)						
Number	1,219	625	99	267		
Age, years	59.1 ± 7.1	58.0 ± 6.5^a^	57.7 ± 6.3	58.0 ± 7.1	0.002	0.034
BMI (kg/m^2^)	22.2 ± 2.0	26.9 ± 1.6^a^	23.6 ± 1.2^a, b^	28.0 ± 2.4^a, b, c^	<0.001	<0.001
Waist-to-height ratio	0.49 ± 0.04	0.56 ± 0.03^a^	0.52 ± 0.03^a, b^	0.57 ± 0.05^a, b, c^	<0.001	<0.001
Mean arterial pressure (mmHg)	89.4 ± 12.7	95.2 ± 12.3^a^	92.0 ± 11.8	97.8 ± 13.4^a, b, c^	<0.001	<0.001
Fasting glucose (mmol/L)	5.5 ± 1.3	5.6 ± 1.1	8.4 ± 3.6^a, b^	7.1 ± 2.4^a, b, c^	<0.001	<0.001
Total cholesterol (mmol/L)	5.28 ± 0.98	5.25 ± 0.96	5.43 ± 1.01	5.29 ± 1.06	0.423	0.366
TG (mmol/L)	1.4 ± 1.27	1.7 ± 0.9^a^	2.3 ± 2.0^a, b^	2.0 ± 1.28^a, b^	<0.001	<0.001
LDL-C (mmol/L)	3.36 ± 0.87	3.42 ± 0.86	3.43 ± 0.94	3.42 ± 0.96	0.390	0.356
HDL-C (mmol/L)	1.27 ± 0.32	1.13 ± 0.24^a^	1.10 ± 0.25^a^	1.07 ± 0.22^a, b^	<0.001	<0.001
TG / HDL-C	1.3 ± 1.6	1.6 ± 1.0^a^	2.4 ± 2.5^a, b^	2.1 ± 1.7^a, b^	<0.001	<0.001
hs-CRP (μg/mL)	2.6 ± 8.0	2.2 ± 3.9	3.2 ± 4.9	3.1 ± 6.4	0.207	0.134
Insulin (mIU/L)	4.6 ± 2.0	6.3 ± 2.1^a^	10.8 ± 4.0^a, b^	13.4 ± 6.5^a, b, c^	<0.001	<0.001
HOMA-IR	1.11 ± 0.51	1.55 ± 0.52^a^	3.7 ± 1.6^a, b^	4.1 ± 2.5^a, b, c^	<0.001	<0.001
25(OH)D (ng/mL)	37.0 ± 19.0	35.3 ± 17.2	32.9 ± 16.8	33.4 ± 18.0^a^	0.005	0.002
Vitamin D deficiency, *n* (%)	261 (21.4)	136 (21.8)	28 (28.3)	76 (28.5)	0.040	0.009

BMI, body mass index; TG, Triglycerides; LDL-C, low-density lipoprotein-cholesterol; HDL-C, high-density lipoprotein-cholesterol; hs-CRP, high-sensitivity C-reactive protein; HOMA-IR, Homeostasis Model Assessment-Insulin Resistance; 25(OH)D, 25-OH-Vitamin D; MHNW, Metabolically healthy normal weight; MHO, metabolically healthy obesity; MUNW, metabolically unhealthy normal weight; MUO, metabolically unhealthy obesity.

a, b, c significant post-hoc comparisons vs. MHNW, MHO, and MUNW, respectively.

Among the four metabolic groups of men aged < 50 years, there were significant incremental trends in TC, TG, insulin, and HOMA-IR levels. The MHO group had the lowest vitamin D level (28.2 ng/mL) and the highest prevalence of vitamin D deficiency (35.8%, Table 2).

Among men aged > 50 years, there was no significant difference in TC, LDL-C, and hs-CRP levels among the four groups. Individuals with MUNW had the highest FBG, TG, and TG/HDL-C levels. Insulin and HOMA-IR levels showed an incremental trend among the four groups. The percentage of vitamin D deficiency also showed an increasing trend among the four groups (*P* = 0.009, Table 2).

### Characteristics of women according to metabolic phenotypes

The women were classified into four groups: MHNW, MHO, MUNW, and MUO. Table 3 shows the characteristics of the women according to the four metabolic phenotypes stratified by age.

**Table 3:** Baseline characteristics of women according to metabolic phenotypes stratified by age.

**Characteristics**	**MHNW**	**MHO**	**MUNW**	**MUO**	***P*** **-value**	***P*** **-trend**
Women < 50 years old (*n* = 960)						
Number	753	125	44	38		
Age, years	40.1 ± 6.3	43.0 ± 5.2^a^	42.0 ± 5.6	44.1 ± 5.4^a^	<0.001	0.001
BMI (kg/m^2^)	21.2 ± 2.1	26.8 ± 1.8^a^	22.6 ± 1.4^a, b^	27.7 ± 1.9^a, c^	<0.001	<0.001
Waist-to-height ratio	0.46 ± 0.041	0.54 ± 0.044^a^	0.49 ± 0.038^a, b^	0.56 ± 0.035^a, b, c^	<0.001	<0.001
Mean arterial pressure (mmHg)	78.0 ± 10.2	85.6 ± 12.7^a^	84.3 ± 9.9^a^	91.4 ± 13.4^a, b, c^	<0.001	<0.001
Fasting glucose (mmol/L)	5.0 ± 0.51	5.2 ± 0.50^a^	5.7 ± 1.0^a, b^	6.0 ± 1.6^a, b^	<0.001	<0.001
Total cholesterol (mmol/L)	4.82 ± 0.85	5.02 ± 1.10	5.04 ± 0.99	5.25 ± 0.88^a^	0.002	0.005
TG (mmol/L)	0.85 ± 0.68	1.15 ± 0.63^a^	1.29 ± 0.59^a^	1.85 ± 1.90^a, b, c^	<0.001	<0.001
LDL-C (mmol/L)	2.87 ± 0.74	3.13 ± 0.86^a^	3.22 ± 0.89^a^	3.26 ± 0.78^a^	<0.001	0.002
HDL-C (mmol/L)	1.48 ± 0.30	1.31 ± 0.27^a^	1.29 ± 0.25^a^	1.22 ± 0.23^a^	<0.001	<0.001
TG / HDL-C	0.64 ± 0.83	0.94 ± 0.67^a^	1.10 ± 0.72^a^	1.7 ± 2.2^a, b, c^	<0.001	<0.001
hs-CRP (μg/mL)	0.89 ± 2.4	1.6 ± 2.4^a^	1.1 ± 1.3	3.1 ± 5.0^a, b, c^	<0.001	<0.001
Insulin (mIU/L)	5.5 ± 2.1	6.9 ± 2.1^a^	11.7 ± 2.2^a, b^	14.3 ± 4.1^a, b, c^	<0.001	<0.001
HOMA-IR	1.24 ± 0.49	1.60 ± 0.51^a^	2.91 ± 0.52^a, b^	3.7 ± 1.2^a, b, c^	<0.001	<0.001
25(OH)D (ng/mL)	26.6 ± 13.3	28.5 ± 12.7	28.4 ± 11.6	22.8 ± 10.5	0.085	0.099
Vitamin D deficiency, *n* (%)	273 (36.3)	39 (31.2)	11 (25.0)	18 (47.4)	0.130	0.917
Women ≥50 years old (*n* = 1,856)						
Number	1,036	502	116	202		
Age, years	57.7 ± 6.3	58.5 ± 6.3	59.7 ± 7.7^a^	59.6 ± 7.0^a^	<0.001	<0.001
BMI (kg/m^2^)	22.1 ± 1.8	27.0 ± 1.9^a^	23.3 ± 1.3^a, b^	28.5 ± 2.6^a, b, c^	<0.001	<0.001
Waist-to-height ratio	0.50 ± 0.041	0.57 ± 0.040^a^	0.53 ± 0.036^a, b^	0.59 ± 0.045^a, b, c^	<0.001	<0.001
Mean arterial pressure (mmHg)	87.2 ± 13.2	93.2 ± 12.8^a^	94.7 ± 12.7^a^	97.7 ± 12.4^a, b^	<0.001	<0.001
Fasting glucose (mmol/L)	5.3 ± 1.0	5.4 ± 0.71	7.0 ± 2.6^a, b^	6.4 ± 1.7^a, b, c^	<0.001	<0.001
Total cholesterol (mmol/L)	5.53 ± 0.99	5.50 ± 0.99	5.59 ± 1.11	5.57 ± 0.98	0.749	0.367
TG (mmol/L)	1.2 ± 0.73	1.4 ± 0.79^a^	1.8 ± 1.3^a, b^	1.9 ± 1.2^a, b^	<0.001	<0.001
LDL-C (mmol/L)	3.42 ± 0.86	3.50 ± 0.85	3.54 ± 1.02	3.53 ± 0.92	0.137	0.089
HDL-C (mmol/L)	1.46 ± 0.32	1.35 ± 0.28^a^	1.27 ± 0.25^a^	1.23 ± 0.27^a, b^	<0.001	<0.001
TG / HDL-C	0.90 ± 0.79	1.13 ± 0.79^a^	1.58 ± 1.46^a, b^	1.65 ± 1.18^a, b, c^	<0.001	<0.001
hs-CRP (μg/mL)	1.8 ± 5.7	2.4 ± 4.2	2.6 ± 7.8	3.0 ± 3.9^a^	0.006	0.005
Insulin (mIU/L)	5.1 ± 1.97	6.6 ± 2.0^a^	12.0 ± 5.6^a, b^	13.4 ± 6.0^a, b, c^	<0.001	<0.001
HOMA-IR	1.2 ± 0.50	1.6 ± 0.49^a^	3.6 ± 1.91^a, b^	3.7 ± 1.87^a, b^	<0.001	<0.001
25(OH)D (ng/mL)	31.9 ± 14.7	31.1 ± 14.6	32.5 ± 13.9	29.3 ± 12.8	0.091	0.075
Vitamin D deficiency, *n* (%)	238 (23.0)	135 (26.9)	22 (19.0)	59 (29.2)	0.066	0.124

BMI, body mass index; TG, Triglycerides; LDL-C, low-density lipoprotein-cholesterol; HDL-C, high-density lipoprotein-cholesterol; hs-CRP, high-sensitivity C-reactive protein; HOMA-IR, Homeostasis Model Assessment-Insulin Resistance; 25(OH)D, 25-OH-Vitamin D; MHNW, Metabolically healthy normal weight; MHO, metabolically healthy obesity; MUNW, metabolically unhealthy normal weight; MUO, metabolically unhealthy obesity.

a, b, c significant posthoc comparisons vs. MHNW, MHO, and MUNW, respectively.

Among the four metabolic groups of women aged < 50 years, the levels of metabolic biomarkers, including FBG, TC, TG, LDL-C, insulin, and HOMA-IR, showed significant incremental trends. The MUO group had the highest MAP level, lowest vitamin D level (22.8 ng/mL), and the highest prevalence of vitamin D deficiency (47.4%). However, there was no significant difference in vitamin D levels and prevalence of vitamin D deficiency among the four groups (*P* = 0.085 and *P* = 0.13, respectively, Table 3).

When considering women aged > 50 years, the MAP, TG, hs-CRP, insulin, and HOMA-IR levels showed significant incremental trends. However, TC and LDL-C levels were not significantly different among the four groups. The MUO group had the lowest vitamin D level (29.3 ng/mL) and the highest prevalence of vitamin D deficiency (29.2%). There was no significant difference in vitamin D levels and prevalence of vitamin D deficiency among the four groups (*P* = 0.091 and *P* = 0.066, respectively, Table 3).

### Association between metabolic phenotypes and vitamin D deficiency

The associations between the metabolic phenotypes and vitamin D deficiency are shown in Table 4. Compared with MHNW, the MHO group showed a higher odds ratio (OR) for vitamin D deficiency in men aged < 50 years, which remained statistically significant after adjusting for cardiometabolic factors, including MAP, TG/HDL-C, and hs-CRP [1.35, 95% confidence interval (CI) 1.05–1.73]. By contrast, in men aged > 50 years, the risk of vitamin D deficiency was greater in the MUO group (OR 1.44, 95% CI 1.05–1.97) followed by MUNW (OR 1.53, 95% CI 0.96–2.43, *P* = 0.076) with borderline significance in multivariable analysis compared with that of the MHNW group (Table 4).

**Table 4:** Association between metabolic phenotypes and vitamin D deficiency stratified by age and sex.

	**Number**	**Vitamin D deficiency**, ***n*** **(%)**	**Univariable analysis**		**Multivariable analysis** ^#^	
			**OR (95% CI)**	***P*** **-value**	**OR (95% CI)**	***P*** **-value**
Men < 50 years old						
MHNW	790	234 (29.6)	1		1	
MHO	492	176 (35.8)	1.32 (1.04–1.68)	0.022	1.35 (1.05–1.73)	0.019
MUNW	65	20 (30.8)	1.06 (0.61–1.83)	0.846	1.08 (0.62–1.89)	0.778
MUO	282	79 (28.0)	0.92 (0.68–1.25)	0.611	0.95 (0.68–1.31)	0.743
Men ≥50 years old						
MHNW	1,219	261 (21.4)	1		1	
MHO	625	136 (21.8)	1.02 (0.81–1.29)	0.863	0.997 (0.784–1.269)	0.982
MUNW	99	28 (28.3)	1.45 (0.92–2.29)	0.114	1.53 (0.96–2.43)	0.076
MUO	267	76 (28.5)	1.46 (1.08–1.97)	0.013	1.44 (1.05–1.97)	0.022
Women <50 years old						
MHNW	753	273 (36.3)	1		1	
MHO	125	39 (31.2)	0.80 (0.53–1.20)	0.275	0.94 (0.61–1.43)	0.757
MUNW	44	11 (25.0)	0.59 (0.29–1.18)	0.134	0.66 (0.33–1.34)	0.253
MUO	38	18 (47.4)	1.58 (0.82–3.04)	0.169	2.33 (1.13–4.81)	0.022
Women ≥50 years old						
MHNW	1,036	238 (23.0)	1		1	
MHO	502	135 (26.9)	1.23 (0.97–1.58)	0.093	1.281 (0.996–1.648)	0.053
MUNW	116	22 (19.0)	0.78 (0.48–1.28)	0.329	0.86 (0.52–1.41)	0.546
MUO	202	59 (29.2)	1.38 (0.99–1.94)	0.058	1.54 (1.08–2.21)	0.018

OR, odds ratio; CI, confidence interval;BMI, body mass index; TG, Triglycerides; HDL-C, high-density lipoprotein-cholesterol; hs-CRP, high-sensitivity C-reactive protein; MHNW, Metabolically healthy normal weight; MHO, metabolically healthy obesity; MUNW, metabolically unhealthy normal weight; MUO, metabolically unhealthy obesity.

# adjusted for mean arterial pressure + TG/HDL-C ratio and hs-CRP.

In women aged < 50 years, the MUO group demonstrated the highest risk for vitamin D deficiency (OR 2.33, 95% CI 1.13–4.81) compared to the MHNW group after adjusting for MAP, TG/HDL-C, and hs-CRP levels. In women aged > 50 years, the MUO group demonstrated the highest risk for vitamin D deficiency (OR 1.54, 95% CI 1.08–2.21) followed by MHO (OR 1.28, 95% CI 0.996–1.648, *P* = 0.053) with borderline significance compared to that of the MHNW group in multivariable analysis.

## Discussion

Our logistic findings implied that in women of all ages and men aged > 50 years, MUO was associated with vitamin D deficiency. Among men aged < 50 years, MHO had the highest OR for vitamin D deficiency. Subcutaneous adipose tissue can store large amounts of fat-soluble vitamin D (57); thus, leading to less vitamin D entering the blood circulation. Greater subcutaneous fat in women, which is related to estrogen, has a greater influence on serum vitamin D concentration than visceral fat tissue (58). Among men aged < 50 years, metabolically unhealthy participants tended to have more visceral fat; the metabolically healthy obese group tended to have more subcutaneous fat, thus leading to the current findings. Lifestyle differences may also contribute to sex and age differences. Younger females generally work indoors and often intentionally avoid sunshine, whereas older male and female might take vitamin D supplements and have more time to engage in outdoor activities (59). To the best of our knowledge, this is a novel study demonstrating cardiovascular risk factors and vitamin D deficiency according to sex, age, and metabolic body composition status in a large Chinese population. The study results provide physicians with useful information regarding vitamin D deficiency and both age- and sex-specific intervention methods to decrease cardiometabolic risk.

A small population study found that obese individuals had significantly lower serum 25-hydroxy vitamin D (25[OH]D) levels than normal-weight participants, regardless of metabolic phenotypes (60). Patchaya et al. performed a retrospective chart review of outpatient medical records. Patients aged > 18 years with BMI > 30 kg/m^2^ were enrolled and divided into two groups: MHO and MUO. They found no significant differences in the 25(OH)D levels between individuals with MHO and MUO. In addition, there was a negative correlation between 25(OH)D levels and adiposity markers (BMI, body weight, and waist circumference), but not between 25(OH)D levels and lipid parameters or HOMA-IR (61). An Iranian population-based study found that 25(OH)D levels were lower in patients with MUO than in those with MHO. Reduced vitamin D concentrations were associated with cardiometabolic and inflammatory markers in MUO compared with MHO. This study did not find a correlation between serum 25(OH)D levels and BMI in obese participants, but it was negatively correlated with waist circumference. Adipose tissue distribution has been hypothesized to be associated with the bioavailability of 25(OH)D (62). Another cross-sectional study recruited 111 healthy adults without diabetes. After adjusting for age, sex, and body fat percentage, 25(OH)D was no longer associated with insulin sensitivity, 2 h glucose, or hs-CRP but remained associated with fasting glucose. The authors interpreted that the association between vitamin D and cardiometabolic risk among healthy adults without diabetes is largely mediated by adiposity (63).

The most commonly mentioned mechanisms explaining the low vitamin D level in individuals with obesity include (i) less sun exposure, (ii) negative feedback from an increased 1,25(OH)D concentration, (iii) sequestration of vitamin D within adipose tissue, and (iv) volumetric dilution resulting in lower 25(OH)D concentration (64).

We also found that for hs-CRP levels, in both sexes aged < 50 years, the highest value was observed in the MUO group, followed by the MHO group, with statistical significance. However, among men aged > 50 years, the MUNW group had the highest hs-CRP level, followed by the MUO group, without statistical significance. Among women aged > 50 years, the MUO group had the highest hs-CRP level, followed by the MUNW group *(P* for trend = 0.005). Hs-CRP, which represents inflammation status, is more influenced by obesity in the younger population, and it is more frequently correlated with metabolically unhealthy status in the older population.

Vitamin D affects adipogenesis, apoptosis, oxidative stress, inflammation, and lipid metabolism (65). Hypponen et al. reviewed the evidence of calcitriol-induced inhibition of many of the adverse effects of obesity. For example, calcitriol suppresses the secretion of pro-inflammatory cytokines, stimulates the secretion of anti-inflammatory cytokines from macrophage-infiltrated adipose tissue, and upregulates insulin growth factor 1 (IGF-1) secretion, which has protective effects against metabolic syndrome. Calcitriol also promotes insulin secretion from islet beta cells, suppresses the overactivity of the renin-angiotensin system in islets, and protects against apoptosis. In obesity-related dyslipidemia, calcitriol can reduce hepatic TG synthesis (66).

Some studies have demonstrated the effects of vitamin D supplementation. A 12-week randomized controlled trial revealed that improvement of vitamin D status in T2DM patients resulted in the amelioration of systemic inflammatory markers, such as hs-CRP and IL-6 (67). A recent meta-analysis demonstrated that vitamin D improves serum levels of TC, TG, and LDL in patients with T2DM (68). A case-control study that focused on men with spinal cord injury demonstrated that even a small increase in vitamin D intake may improve TC independent of lean mass. Vitamin D adjusted for total dietary intake, was positively correlated with carbohydrate profile parameters (69). Another Mendelian randomization study found that a 25 nmol/L higher concentration was associated with a 14% lower risk of T2DM (70). Wenclewska et al. found that among patients suffering from metabolic disturbances and T2DM, supplementation with 2,000 IU vitamin D for 3 months reduced the level of oxidative deoxyribonucleic acid (DNA) damage, HOMA-IR, and TG/HDL ratio (71).

Our study focused on a large Asian population, stratified by age and sex. We evaluated cardiometabolic biomarkers and the risk of vitamin D deficiency among the four metabolic phenotypes. We combined both HOMA-IR and metabolic syndrome criteria to define metabolic health or unhealthy status, which makes it more indicative of morbidity and mortality and provides relatively convincing results.

Nevertheless, this study has several limitations. First, the cross-sectional study design makes it impractical to establish causal relationships. Second, we did not record participants' lifestyles, including physical activity, sun exposure, dietary habits, and use of vitamin D supplements. Third, our study participants were relatively healthy or had better health awareness; therefore, they may not represent the general population. Further research is warranted to elucidate the potential protective or anti-inflammatory effects of vitamin D in different obesity phenotypes.

In conclusion, in a relatively healthy population, our data revealed that in women of all ages and men aged > 50 years, the MUO group had the highest OR for vitamin D deficiency. Among men aged < 50 years, the highest OR for vitamin D deficiency was observed in the MHO group. The inflammatory biomarker hs-CRP is more strongly correlated with obesity in younger adults, and it is more correlated with metabolically unhealthy status in older individuals.

## Data availability statement

The original contributions presented in the study are included in the article/supplementary material, further inquiries can be directed to the corresponding author.

## Ethics statement

The studies involving human participants were reviewed and approved by Institutional review board of Chang Gung Memorial Hospital. Written informed consent for participation was not required for this study in accordance with the national legislation and the institutional requirements.

## Author contributions

Y-CC and W-CL conceived the study concept, design and contributed equally to this study. WY, H-YH, and X-JX were responsible for data collection. Y-CC drafted the manuscript. W-CL and J-YC interpreted the data. J-YC revised the final manuscript. All the authors approved the final version of the manuscript.

## Conflict of interest

The authors declare that the research was conducted in the absence of any commercial or financial relationships that could be construed as a potential conflict of interest.

## Publisher's note

All claims expressed in this article are solely those of the authors and do not necessarily represent those of their affiliated organizations, or those of the publisher, the editors and the reviewers. Any product that may be evaluated in this article, or claim that may be made by its manufacturer, is not guaranteed or endorsed by the publisher.
